# Strawberry notch homolog 2 is a novel inflammatory response factor predominantly but not exclusively expressed by astrocytes in the central nervous system

**DOI:** 10.1002/glia.22841

**Published:** 2015-04-22

**Authors:** Magdalena Grill, Taylor E. Syme, Aline L. Noçon, Andy Z. X. Lu, Dale Hancock, Stefan Rose‐John, Iain L. Campbell

**Affiliations:** ^1^ School of Molecular Bioscience University of Sydney Sydney Australia; ^2^ Institute of Experimental and Clinical Pharmacology, Medical University of Graz Graz Austria; ^3^ Department of Biochemistry University of Kiel Kiel Germany

**Keywords:** glycoprotein 130, strawberry notch homolog 2, astrocyte, inflammation, cytokines

## Abstract

Interleukin‐6 (IL‐6) participates in the host response to injury and infection in the central nervous system (CNS). We identified strawberry notch homolog 2 (*Sbno2*) as an IL‐6‐stimulated gene in murine astrocytes. *Sbno2* is a mouse homolog of the sno gene in Drosophila but little is known about the regulation or function of the mammalian gene. Here we examined the regulation of the *Sbno2* gene in astrocytes *in vitro* and in the murine CNS following systemic endotoxin administration. In murine and human cultured astrocytes, *Sbno2* gene expression was significantly upregulated in a dose‐ and time‐dependent fashion by hyper‐IL‐6 (IL‐6 + soluble IL‐6 receptor). The level of Sbno2 mRNA was also upregulated significantly in murine astrocytes by other glycoprotein130 cytokine‐family members and the pro‐inflammatory cytokines interleukin‐1 beta and tumor necrosis factor alpha. These changes were reflected by corresponding alterations in the level of the SBNO2 protein. Inhibiting protein synthesis resulted in higher Sbno2 mRNA and did not abolish the upregulation of Sbno2 mRNA mediated by hyper‐IL‐6. Inhibition of transcription led to a rapid reduction in hyper‐IL‐6‐induced Sbno2 mRNA in astrocytes suggesting that the Sbno2 mRNA is quite unstable. Following intra‐peritoneal lipopolysaccharide injection in mice, Sbno2 mRNA levels in the brain were significantly increased. Cellular localization studies revealed that this increase in Sbno2 mRNA occurred predominantly in astrocytes and in the choroid plexus and in some microglia, endothelial cells, and neurons. These findings are consistent with SBNO2 functioning as an acute inflammatory response gene in astrocytes as well as other cells in the CNS. GLIA 2015;63:1738–1752

## Introduction

Astrocytes are highly plastic cells of the central nervous system (CNS) that are implicated in the pathogenesis of various CNS disease states (Sofroniew, 2009). Upon injury or infection, astrocytes become activated and undergo a wide spectrum of progressive molecular and cellular changes that give rise to an altered functional phenotype (Sofroniew, 2009). Among the molecular mediators triggering reactive astrogliosis is the gp130 family of cytokines (Damiani and O'Callaghan, 2007; Sriram et al., 2004). This includes the pleiotropic cytokine interleukin (IL)‐6, as demonstrated in transgenic mice with astrocyte targeted IL‐6 production (Campbell et al., 1993; Chiang et al., 1994). IL‐6 first achieved attention in the CNS due to its dysregulation in autoimmune disease and neurological disorders (Frei et al., 1991; Mogi et al., 1994; Wood et al., 1993). Nevertheless, it has been shown to have both pro‐ as well as anti‐inflammatory actions and there are many examples of detrimental as well as beneficial effects of IL‐6 (Scheller et al., 2011; Spooren et al., 2011).

IL‐6 binds to the membrane bound IL‐6 receptor (IL‐6R) which is unable to signal alone but triggers intracellular signal transduction through oligomerization with the signalling co‐receptor glycoprotein (gp) 130 [reviewed by Ernst and Jenkins (2004)]. This receptor subunit is also used by several related cytokines including IL‐11, leukemia inhibitory factor (LIF), oncostatin M (OSM), cardiotrophin‐like cytokine (CLC), cardiotrophin‐1 (CT‐1), ciliary neurotrophic factor (CNTF), neuropoietin (NPN), and IL‐27. In addition to the interaction with the membrane bound IL‐6R, known as classic signaling, IL‐6 also binds to the soluble (s) IL‐6R that is generated by proteolysis and shedding of the extracellular domain of the plasma membrane protein or by alternative mRNA splicing (Lust et al., 1992; Mullberg et al., 1993). In the presence of the ligand, IL‐6, sIL‐6R is agonistic, stimulating cells that express gp130 but that lack the membrane bound form of IL‐6R. This process is called trans‐signaling (Rose‐John and Heinrich, 1994) and is associated with mainly pro‐inflammatory effects, while classic signaling is thought to be involved in mainly physiological functions [reviewed by Rose‐John (2012) and Scheller et al. (2011)]. A synthetic fusion protein of IL‐6 and its sIL‐6R called hyper‐IL‐6 is widely used to experimentally mimic the effects of trans‐signaling (Fischer et al., 1997; Jones et al., 2005; Rose‐John et al., 2006). With regard to the CNS, *in vitro* studies have shown that astrocytes and neurons respond mainly to IL‐6 trans‐signaling rather than classical signaling (März et al., 1998, 1999; Van Wagoner et al., 1999). Blocking of trans‐signaling *in vivo* ameliorates many of the detrimental actions of IL‐6 in the brain (Campbell et al., 2014 ).

Activation of gp130 induces signal transduction via two main pathways: the Janus kinases/signal transducers and activators of transcription (JAK/STAT) pathway and the SH‐domain containing tyrosine phosphatase 2/rat sarcoma/extracellular signal‐regulated kinases (SHP2/Ras/ERK) mitogen‐activated protein kinases (MAPK) pathway [reviewed by Ernst and Jenkins (2004)]. Negative feedback mechanisms to keep these signaling pathways under control are mediated by SHP2 as well as suppressor of cytokine signaling (SOCS) proteins (Croker et al., 2008, 2003; Lang et al., 2003). Transcriptional induction of SOCS, especially SOCS3, is induced via the IL‐6/gp130 activated JAK/STAT pathway.

Astrocytes are both producer and responder cells to IL‐6. The pleiotropic role of IL‐6 on astrocytes was described in detail in recent reviews (Erta et al., 2012; Spooren et al., 2011). In differentiated glial cells, IL‐6 induced astrogliosis may be mediated via JAK‐2/STAT3 without involvement of ERK and STAT1 (Damiani and O'Callaghan, 2007; Sriram et al., 2004). STAT3 mediated reactive astrogliosis is associated with improved recovery after injury (Herrmann et al., 2008). Furthermore, IL‐6 increases the production of inflammatory mediators, induces the production of growth factors, and enhances chemotaxis of astrocytes (Barnum et al., 1996; Chikuma et al., 2009; März et al., 1999; Odemis et al., 2002; Quintana et al., 2008).

To better understand the impact of IL‐6 on astrocytes, transcriptomic studies were undertaken by us (Frausto and Campbell, unpublished), that identified *Sbno2* as a novel and highly upregulated IL‐6 target gene. The SBNO2 protein is a putative DExD/H‐box containing helicase and one of the two known mouse homologs of the Drosophila gene sno (Marchler‐Bauer et al., 2011; Takano et al., 2010, 2011). Sno is involved in EGFR and Notch‐related signaling and consequential cell fate determination during development (Coyle‐Thompson and Banerjee, 1993; Majumdar et al., 1997; Tsuda et al., 2002). Takano et al. (2010, 2011) reported the involvement of the *strawberry notch* family genes *sbno1* and *sbno2a* in zebrafish brain development. The mouse homolog *Sbno1* is upregulated in neurons upon Reelin signaling (Baba et al., 2006). The Reelin pathway interacts with the Notch pathway (Hashimoto‐Torii et al., 2008), also linking the murine SBNO1 to Notch signaling. Both pathways may be involved in neurogenesis and neuronal migration. Interestingly however, it was reported that IL‐10 stimulates the expression of the *Sbno2* gene in a STAT3‐dependent fashion in bone marrow derived macrophages (BMDMs) (El Kasmi et al., 2007). This study also implicated SBNO2 in the anti‐inflammatory response (AIR) via inhibition of NFκB signaling. However, a more recent report in which SBNO2 was linked to osteoclast function failed to confirm NFκB pathway inhibition by SBNO2 (Maruyama et al., 2013). Other than these limited studies very little is known about the regulation of SBNO2 production or the function of this protein in mammalian cells and especially in the CNS.

Here, we hypothesize that SBNO2 is a novel downstream regulator of IL‐6/gp130 and other cytokine actions in the brain. The present study was aimed at investigating the regulation of the *Sbno2* gene in astrocytes by different cytokines *in vitro* as well as the regulation and cellular localization in the murine brain in a model for endotoxin‐induced systemic inflammation.

## Materials and Methods

### Cytokines

Recombinant hyper‐IL‐6 was prepared as described previously (Fischer et al., 1997). Endotoxin‐free, recombinant murine OSM was from R&D Systems (Minneapolis, MN), recombinant murine tumor necrosis factor alpha (TNFα) and IL‐11 were from Peprotech (Rocky Hill, NJ), recombinant murine IL‐1β from Cell Signaling (Danvers, MA), and recombinant murine LIF from Millipore (Temecula, CA). Lipopolysaccharide (LPS) from *E. coli* serotype 026:B6 was from Sigma‐Aldrich (St. Louis, MO).

### Antibodies

For immunoblotting: antibodies produced in rabbit against murine SBNO2, C‐terminal peptide (1:640), were custom prepared by Biomatik (Cambridge, ON, Canada). Antibodies against STAT3 (1:1,000), phospho‐STAT3 (Tyr705, 1:2,000), phospho‐p44/42 MAPK ((phospho)‐ERK 1/2) (Thr 202/Tyr 204, 1:3,000), NFκB (p65, 1:3,000), phospho‐NFκB (p65, Ser536, 1:1,000) all produced in rabbit were from Cell Signaling. The ERK 1/2 antibody (1:10,000, host: rabbit) was from Sigma‐Aldrich, while GAPDH antibody (1:100,000, host: mouse) was from Millipore. Horse raddish peroxidase (HRP)‐coupled goat anti‐rabbit IgG (SC2004, Santa Cruz Biotechnology Inc., Dallas, TX) or goat anti‐mouse IgG (Fc specific, A0168, Sigma‐Aldrich) secondary antibodies were used for detection.

For flow cytometry: CD11b‐APC (1:200, eBioscience, San Diego, CA) and GLAST‐PE (1:11, Miltenyi Biotec, Bergisch Gladbach, Germany) antibodies plus corresponding isotype controls were used.

### Animals

C57BL/6 mice were either obtained from ARC (Canning Vale, Western Australia) or bred in‐house. All animal research involved in this project received approval from the University of Sydney Animal Ethics Committee and complied with the Australian Government regulations as outlined by the National Health and Medical Research Council (NHMRC).

### Cell Culture

Primary murine cell cultures of highly purified astrocytes were prepared from 5 to 10 brains of 1‐ to 3 day‐old C57BL/6 mice as described previously (Carter et al., 2007). Primary astrocytes were maintained in Dulbecco's modified eagle medium (D‐MEM) supplemented with 10% fetal bovine serum (FBS) and penicillin (100 units/mL)/streptomycin (100 μg/mL) (all from Gibco®, Life Technologies Corporation, Carlsbad, CA) and cultured in poly‐d‐lysine coated cell culture flasks (BD Biosciences).

After magnetic‐activated cell sorting (MACS, Miltenyi), the purity of the cultures was confirmed using CD11b‐APC (microglia marker) and GLAST‐PE (astrocyte marker) antibodies and quantified by flow cytometry using FACScalibur (BD Biosciences, North Ryde, NSW, Australia) and Flow‐Jo Software (Tree Star, Stanford, CA). By this approach astrocyte cultures routinely were found to contain < 0.2% contamination with microglia. Purified astrocytes were seeded at 5 × 10^5^ cells/well into 6‐well plates (BD Biosciences). Thereafter, each well was handled as an individual sample. Cells were further cultured for two days. Before treatment, primary astrocytes were washed with 1× phosphate buffered saline (PBS) and starved in serum‐free D‐MEM overnight (12 − 16 hours (h)).

Human astrocytes (ScienCell Research Laboratories, San Diego, CA) were kept in D‐MEM (high glucose) with GlutaMAX™, 10% FBS, 1× N2‐supplement and penicillin (100 units/mL)/streptomycin (100 μg/mL) (all reagents from Gibco®, according to the manufacturers recommendations for maintenance of primary human astrocytes). Cells were seeded at 5 × 10^5^ cells/well into 6‐well plates or 1.5 × 10^6^ cells into T25 cell culture flasks (BD Biosciences) and cultured for two to three days. Before treatment, human astrocytes were washed with 1× PBS and cultured overnight (12 − 16 h) in serum‐ and supplement‐free D‐MEM.

### Cell Culture Treatments

Primary murine astrocyte cultures were treated with hyper‐IL‐6 at different concentrations (0, 1, 5, 25, 50, 100 ng/mL) for 2 h. Primary murine astrocytes as well as human astrocytes were treated with 25 ng/mL of hyper‐IL‐6 for different times (2, 6, 12, 24 h). All other cytokines were used at concentrations of 25 ng/mL for 2 h treatment of cells for RNA or 6 h treatment for protein extraction. Cycloheximide (CHX, Sigma‐Aldrich) was used at 10 μg/mL and added to medium with or without hyper‐IL‐6 (25 − 50 ng/mL, the concentration was increased in a repeat due to batch differences) and cells were treated for 2, 4, 6, and 8 h. To study the effects of actinomycin D (ActD, Sigma‐Aldrich), cells were treated with 25 ng/mL hyper‐IL‐6 in D‐MEM. After 2 h, 5 μl of ActD in DMSO (final concentration: 10 μg/mL) or 5 μL of DMSO alone (solvent control) were added and incubated for the indicated times.

### LPS‐Induced Endotoxemia Model

The LPS‐endotoxemia model used was described previously (Ip et al., 2011; Stalder et al., 1997). Briefly, C57BL/6 mice received staggered, intra‐peritoneal (i.p.) injections of LPS (2 mg/kg) in sterile PBS. Mice received dual injections given 16 h apart and were euthanized 4 h or 24 h after the second injection. For extraction of RNA, brains were immediately resected and stored at −80 °C pending RNA isolation (see below).

### RNA Isolation and RNase Protection Assay

Total RNA from untreated and cytokine‐treated murine and human astrocytes, as well as from brain, was isolated by Tri‐Reagent (Sigma), using a variation of the method by Chomczynski and Sacchi (1987), according to the manufacturer's protocol.

Generation of RNase protection assay (RPA) probes was performed as described previously (Stalder et al., 1999). Specific sequences used: *Sbno2*: Accession No: NM_183426.1, target sequence: 1421–1725, product length: 305 bp. *Socs3*: Accession No: U88328, target sequence: 361–555, product length: 194 bp. As an internal control, the ribosomal protein (RP) *L32* was used as previously described (Maier, 2002).

Briefly, target sequences were synthesized by reverse transcription and polymerase chain reaction (PCR) and cloned into the pGEM‐4Z vector (Promega, Madison, WI). Cloned pGEM‐4Z plasmids were linearized and used for preparation of RPA probe sets that were further employed for the synthesis of labeled RNA probes. RNA (1–3 μg) samples isolated from cells were hybridized with [^32^P]‐UTP‐labeled probe sets containing the corresponding cRNA probes. Remaining unhybridized RNA was digested by a single‐strand specific RNase. RNA hybrids were denatured and separated, and labeled probes were detected by polyacrylamide gel electrophoresis. A fragment of the RPL32 gene was used as loading control for all probe sets. For quantification, autoradiographs were scanned and analyzed by densitometry using Image‐J (NIH Image software). The corresponding densitometric value for each transcript was expressed as ratio to the RPL32 RNA value.

### Reverse Transcription and Quantitative PCR

Complementary (c)DNA from each RNA preparation of human astrocytes was synthesized with Reverse Transcriptase (Invitrogen SuperScript III™) according to the manufacturer's protocol, using random hexamers as primers. qPCR was performed on these cDNA samples using SybrGreen (FAST SybrGreen master mix, Applied Biosystems) following a two‐step thermocycler program (5 min at 50 °C, 95 °C for 5 min followed by 40 cycles of 3 s at 95 °C then 30 s at 60 °C). Specific primers used: human 18S: Accession no: K03432, primer sequences forward: 5′‐CGCCGCTAGAGGTGAAATTC‐3′, reverse: 5′‐CATTCTTGGCAAATGCTTTCG‐3′; human SBNO2 (recognizes both transcripts): accession no: NM_014963.2, NM_001100122.1, primer sequences forward: 5′‐ATGGCCGACGTCAGCAGCAG‐3′, reverse: 5′‐ATCTTGATGCCCACTTGCTT‐3′. A melt curve was performed subsequently on all reactions. Samples were normalized to 18S internal control.

### Sodium Dodecyl Sulfate‐Polyacrylamide Gel Electrophoresis and Western Blotting

Cells were homogenized in RIPA lysis buffer (50 mM Tris‐HCl (pH 7.5), 150 mM NaCl, 1 mM EDTA, 1% (wt/vol) deoxycholic acid, 1% (vol/vol) triton‐X, 0.1% (wt/vol) sodium dodecyl sulfate (SDS), 2 mM PMSF, 50 mM NaF) containing freshly added protease and phosphatase inhibitor cocktails (1:100, Calbiochem, Sydney, Australia). Total protein levels were measured by a BCA protein assay (Pierce, ThermoScientific, NSW, AU) and protein (5–7 μg) fractionated by SDS‐PAGE and analyzed by immunoblotting as described previously (Sanz et al., 2008; Wang et al., 2002) to determine the levels of SBNO2, STAT3, phospho‐STAT3, ERK 1/2, phospho‐ERK 1/2, NFκB, phospho‐NFκB and GAPDH protein levels. HRP‐coupled anti‐rabbit or anti‐mouse secondary antibodies and Immune star HRP detection kit (Bio‐Rad Laboratories Pty. Ltd., Gladesville, NSW, Australia) were used for detection. Quantification of band intensities was performed by densitometry using Image‐J and densities were normalized to the internal control GAPDH.

### Antibody Peptide‐Blocking

Anti‐SBNO2 antibody (6 μg) and corresponding SBNO2 C‐terminal immunizing peptide (30 μg) were combined in 1× PBS (100 μL), mixed and incubated for 24 h with gentle agitation at 4°C. In parallel, as a positive control, anti‐SBNO2 antibody without immunizing peptide was used at the same concentration and for the same procedure. After centrifugation for 15 min at 18,000*g*, the supernatants were used for Western blot as described above.

### *In Situ* Hybridization‐Immuno‐/Histochemistry

Linearized plasmids containing the target sequence were prepared as described for RPA. For *in situ* hybridization (ISH) a specific Sbno2 cRNA (Accession No: NM_183426.1, target sequence: 1,181–2,124; product length 944 bp) probe was synthesized and cloned in pGEM‐4Z as described above.

Mice were perfused with 20 mL 1× PBS, followed by 20 mL 4% paraformaldehyde in 1× PBS. Brains were isolated, post‐fixed in 4% paraformaldehyde in 1× PBS overnight, dehydrated and embedded in paraffin. ISH with [^33^P]‐UTP‐labeled antisense or sense Sbno2 cRNA probes was done as described previously (Campbell et al., 1994).

To identify Sbno2 mRNA positive cells, immunohistochemistry or histochemistry (IHC/HC) was performed using rabbit anti‐cow GFAP antibody (DAKO) and monoclonal mouse anti‐mouse NeuN (Millipore), or biotinylated tomato lectin (Sigma‐Aldrich). Visualization of bound antibody or lectin was performed using a Vectastain ABC kit and corresponding biotinylated anti‐rabbit or anti‐mouse secondary antibodies contained in the kit and diaminobenzidine/peroxidase (DAB) substrate kit (all Vector Laboratories), performed according to the manufacturer's instructions.

After dehydration through a graded alcohol series and air‐drying, an autoradiography film (Kodak® Biomax® Maximum Resolution Autoradiography Film, Carestream Health, VWR International, Vienna, Austria) was exposed to the slides for 4 days. Thereafter, slides were dipped in NTB Emulsion, Carestream Health (VWR International) and stored in the dark for 2–3 weeks, after which time the slides were developed, counterstained with Mayer's hematoxylin and examined by bright field microscopy.

### Statistical Analysis

For statistical analysis Graphpad Prism 5 Software was used. Excluded outliers were identified using Grubb's outlier test. In general, ANOVA analyses with Dunnet's or Tukey's post‐tests were used to compare multiple treatment groups as indicated. Two‐way ANOVA with Bonferoni's post‐test was used to compare two time courses ± CHX treatment. Results are shown as mean ± SEM as indicated, *P*‐values <0.05 were considered significant (*P < 0.05, **P < 0.01, ***P < 0.001).

## Results

### *Sbno2* mRNA Levels Are Regulated in a Dose‐ and Time‐Dependent Manner in Astrocytes

**Figure 1:**
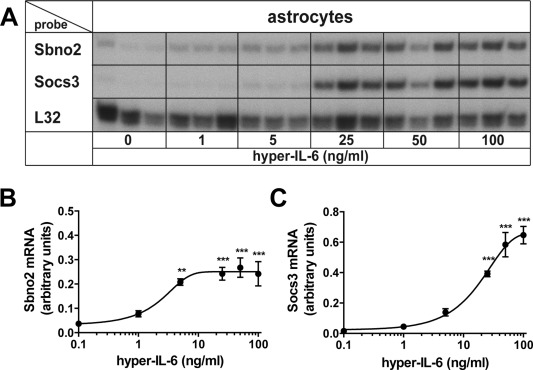
Dose‐dependent regulation of *Sbno2* gene expression by hyper‐IL‐6 in astrocytes. Primary astrocytes were treated with 0, 1, 5, 25, 50, or 100 ng/mL of hyper‐IL‐6, cells were harvested after 2 h and total RNA was isolated and analyzed by RPA. **A**: A representative RPA analysis is shown. The graphs show mean ± SEM of integrated density of (**B**) Sbno2 or (**C**) Socs3 mRNA bands relative to the control RPL32 of two independent RPAs, each 2–3 samples per group (total *n* = 5–6). *P < 0.05, **P < 0.01, ***P < 0.001 compared by ANOVA and Tukey's post test.

**Figure 2:**
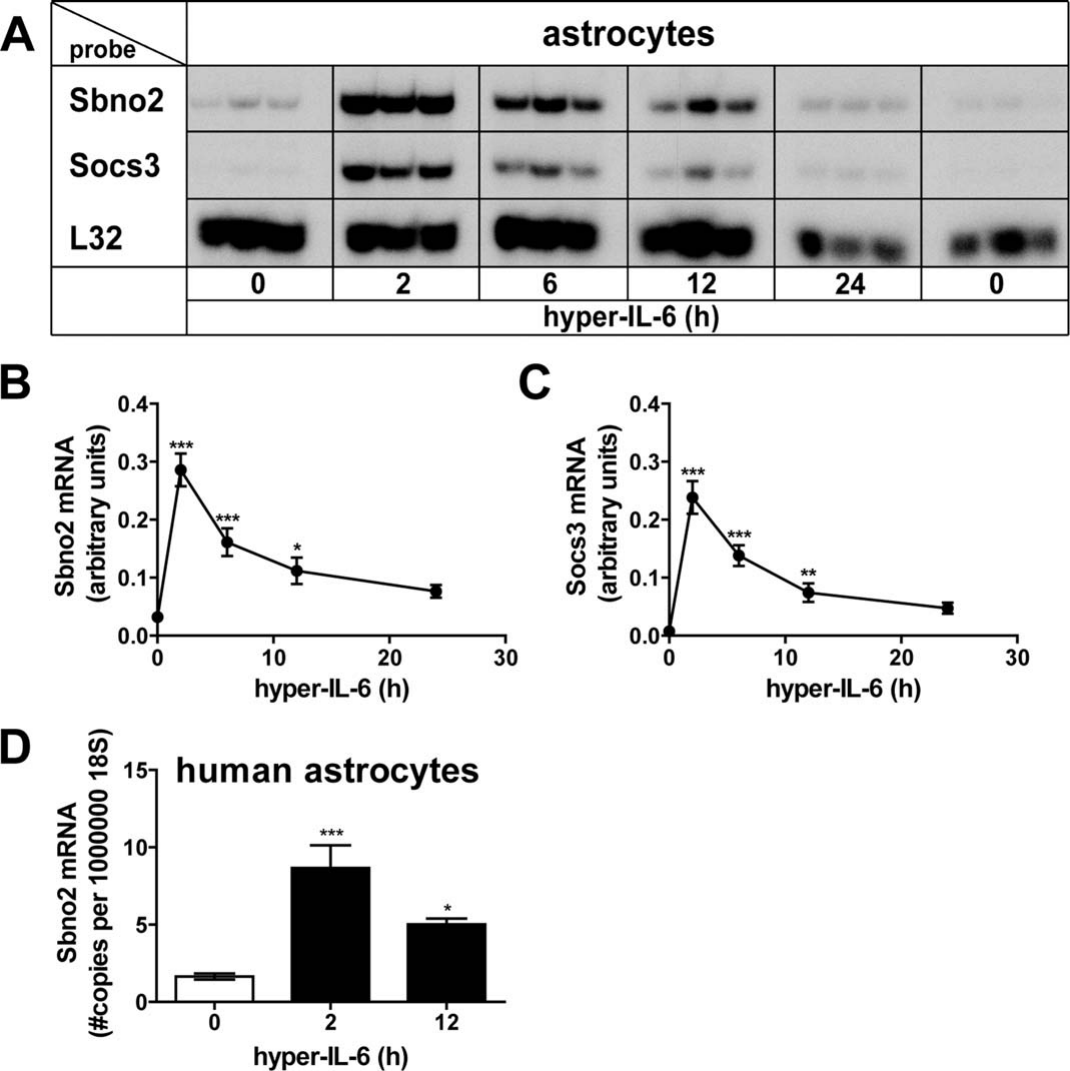
Time‐dependent regulation of *Sbno2* gene expression by hyper‐IL‐6 in murine and human astrocytes. Primary murine astrocytes (**A**–**C**) or primary human astrocytes (**D**) were treated with or without 25 ng/mL hyper‐IL‐6. Cells were harvested at the indicated time points and total RNA was isolated and analyzed by RPA (A–C) or qPCR (D). **A**: A representative RPA is shown. Graphs show mean ± SEM of integrated density of Sbno2 (**B**) or Socs3 (C) mRNA bands relative to the control RPL32 of two independent RPAs, each 2–3 samples per group (total *n* = 5–6). *P < 0.05, **P < 0.01, ***P < 0.001 compared by ANOVA and Tukey's post test. D: The graph shows means ± SEM of three independent qPCR runs with each 1–3 samples per group (total *n* = 7). Values are Sbno2 mRNA copy numbers per 10^6^ 18S (internal control). *P < 0.05, **P < 0.01, ***P < 0.001 compared by ANOVA and Tukey's post test.

Because astrocytes lack IL‐6R and are therefore unresponsive to IL‐6 (März et al., 1999; Van Wagoner et al., 1999), we chose to stimulate astrocytes with hyper‐IL‐6. Initial microarray analysis of hyper‐IL‐6‐treated primary astrocytes (Frausto and Campbell, unpublished) identified novel IL‐6‐regulated target genes in these cells. In parallel to *Socs3*, a well‐known IL‐6 target gene, the expression of the *Sbno2* gene was found to be significantly increased in astrocytes. In order to confirm the microarray results and further clarify the nature of the regulation of the *Sbno2* gene, we examined the responses of murine primary astrocytes to hyper‐IL‐6 to different concentrations or times of treatment. There was a concentration‐dependent increase of Sbno2 and Socs3 mRNAs in response to a 2‐h treatment with hyper‐IL‐6 (Fig. 1A–C). Sbno2 mRNA levels in astrocytes were increased significantly with 5 ng/mL hyper‐IL‐6 and did not increase further with higher concentrations (Fig. 1B). Socs3 mRNA was upregulated significantly with 25 ng/mL and further increased to a maximum with 50 ng/mL (Fig. 1C). The estimated EC50 for hyper‐IL‐6 induction was 2.3 ng/mL for the *Sbno2* gene and 23.4 ng/mL for the *Socs3* gene. Hyper‐IL‐6 (25 ng/mL) also stimulated a time‐dependent increase in Sbno2 mRNA (Fig. 2A,B). Upregulation of Sbno2 (Fig. 2B) and Socs3 (Fig. 2C) mRNA peaked at 2 h and decreased thereafter but remained significantly elevated out to 12‐h treatment time (Fig. 2A–C). Similar to murine astrocytes, Sbno2 mRNA was also significantly upregulated in human astrocytes at 2 and 12 h following treatment with hyper‐IL‐6 (Fig. 2D). In summary, these findings indicate that hyper‐IL‐6 is a potent stimulus for *Sbno2* gene expression in murine and human astrocytes.

### *Sbno2* mRNA Levels Are Regulated by Other IL‐6/Gp130 Cytokines and Pro‐inflammatory Cytokines in Astrocytes

**Figure 3:**
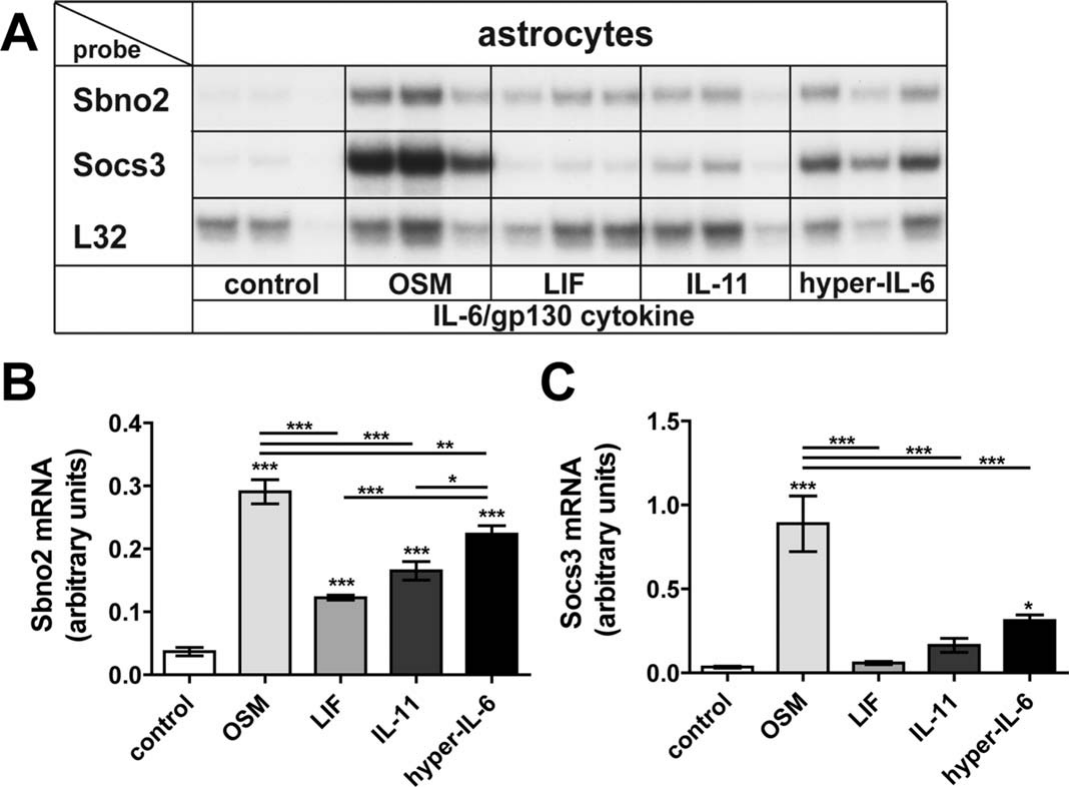
Regulation of *Sbno2* gene expression by other IL‐6/gp130 cytokine family members in murine astrocytes. **A**: *Sbno2* gene expression in astrocytes is induced by OSM, LIF, IL‐11, and hyper‐IL‐6 (each at 25 ng/mL for 2 h), whereas *Socs3* gene expression is significantly induced by OSM and hyper‐IL‐6 only. A representative RPA is shown. Graphs show mean ± SEM of integrated density of Sbno2 (**B**) or Socs3 (**C**) mRNA bands relative to the control RPL32 of two independent RPAs, each 2–3 samples per group (total *n* = 5–6). *P < 0.05, **P < 0.01, ***P < 0.001 compared by ANOVA and Tukey's post test.

Hyper‐IL‐6 signals via the co‐receptor gp130 and JAK/STAT pathway which is also used by other IL‐6/gp130‐like family members. Therefore, we asked whether OSM, LIF, and IL‐11 can regulate *Sbno2* and *Socs3* gene expression. Upon treatment of murine astrocytes with 25 ng/mL of each cytokine for 2 h, Sbno2 mRNA was highly upregulated by OSM and to a significantly lesser degree by LIF, IL‐11, and hyper‐IL‐6 (Fig. 3A,B). Hyper‐IL‐6 was a stronger inducer of *Sbno2* gene expression than LIF or IL‐11. Socs3 mRNA (Fig. 3C) was significantly elevated by OSM or hyper‐IL‐6, with OSM being a more potent inducer than hyper‐IL‐6. Socs3 mRNA was slightly, but not significantly increased by IL‐11 while there was no change with LIF treatment. In all, these findings indicated that in addition to hyper‐IL‐6, other members of the IL‐6/gp130 family also stimulated *Sbno2* gene expression with OSM being the most potent.

**Figure 4:**
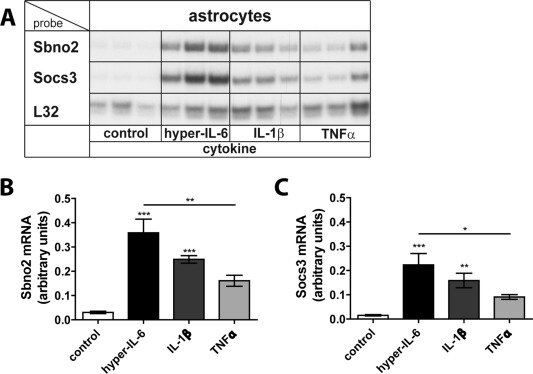
Regulation of *Sbno2* gene expression by other pro‐inflammatory cytokines in astrocytes. **A**: *Sbno2* and *Socs3* gene expression in astrocytes was regulated by IL‐1β and a trend was also seen by TNFα. A representative RPA is shown. Graphs show mean ± SEM of Sbno2 (**B**) or Socs3 (**C**) mRNA bands relative to the control RPL32 of two independent RPAs, 2–6 samples per group (total *n* = 5–9). *P < 0.05, **P < 0.01, ***P < 0.001 compared by ANOVA and Tukey's post test.

We next tested whether other pro‐inflammatory cytokines were able to regulate Sbno2 and Socs3 mRNA levels in these cells (Fig. 4). In addition to hyper‐IL‐6, IL‐1β significantly upregulated Sbno2 mRNA (Fig. 4A,B) and Socs3 mRNA (Fig. 4A,C). An increase in Sbno2 and Socs3 mRNA levels was also found after TNFα treatment, although not significant when compared by one‐way ANOVA and Tukey's post‐test. These data show that *Sbno2* gene expression is not only regulated by the gp130‐family cytokines but also by other pro‐inflammatory cytokines.

### Regulation of *Sbno2* Gene Expression Is Reflected by Corresponding Protein Levels

We next determined whether the cytokine‐mediated changes observed in *Sbno2* gene expression were translated into the corresponding protein. Western blot analysis was performed using a custom‐made antibody that reacted with the C‐terminal region of the murine SBNO2.

**Figure 5:**
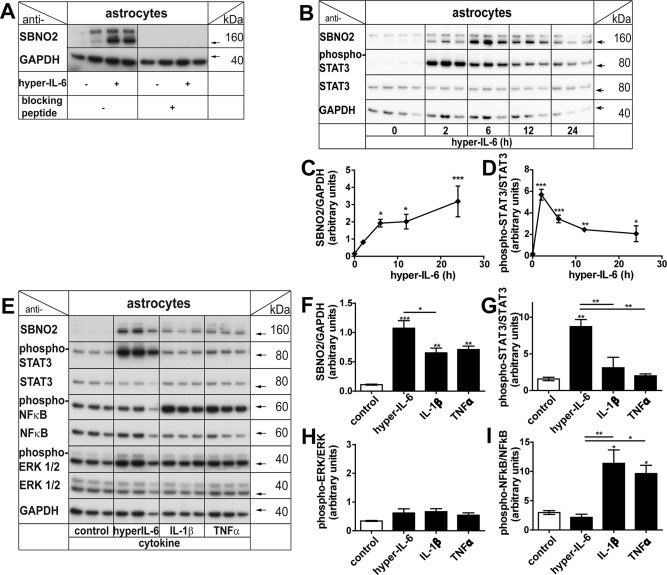
Alterations in *Sbno2* gene expression are also reflected by SBNO2 protein levels. **A**: An antibody against the C‐terminal end of murine SBNO2 revealed 2–3 bands between 160 and 175 kDa. The smaller 1–2 bands were regulated by hyper‐IL‐6 treatment. Incubation of anti‐SBNO2 antibody with blocking peptide eliminated bands that were present when using primary antibody alone. **B**, **C**: In murine astrocytes, treatment with 25 ng/mL hyper‐IL‐6 revealed altered protein levels of SBNO2 that reached significance at 6 h and remained elevated up to 24 h compared with control. B, **D**: Phospho‐STAT3 levels peaked at 2 h. **E**, **F**: Altered SBNO2 protein levels were also found upon 6 h treatment with 25 ng/mL of IL‐1β or TNFα. While hyper‐IL‐6 lead to increased phospho‐STAT3 (**G**), IL‐1β and TNFα showed elevated phospho‐NFκB levels (**I**) compared with control. Phospho‐ERK levels were not significantly altered in any of the groups (**H**). Shown is a representative Western blot and quantification of three to four samples per group (*n* = 3–4). *P < 0.05, **P < 0.01, ***P < 0.001 compared by ANOVA and Dunnet's (C, D) or Tukey's (F–I) post test.

In untreated cells, the antibody recognized two protein bands, one at ∼160 kDa and one at ∼175 kDa (Fig. 5A). In hyper‐IL‐6‐treated cells, the level of the protein band at ∼175 kDa did not change while that at ∼160 kDa increased and an additional band at ∼165 kDa was also detected. Following the addition of an excess of immunizing peptide to the primary antibody, no detectable protein bands were observed confirming the specificity of the antibody. Thus, these studies identified at least two isoforms of murine SBNO2, a constitutive long‐form and a hyper‐IL‐6‐inducible short‐form.

**Figure 6:**
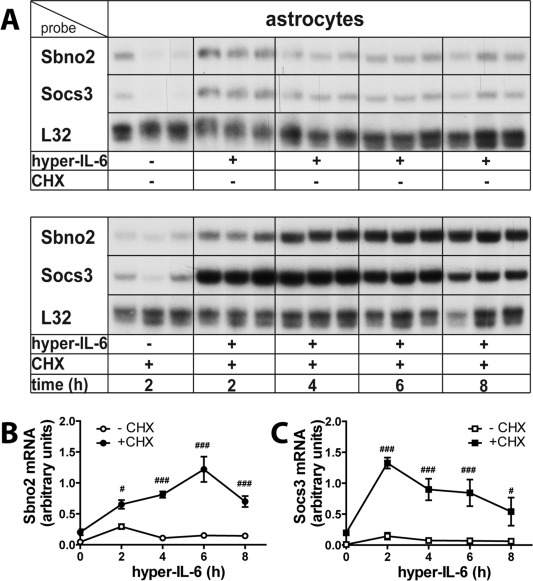
The *Sbno2* and *Socs3* genes are direct response genes to hyper‐IL‐6 in astrocytes. **A**–**C**: Inhibition of protein synthesis by cycloheximide (CHX, 10 μg/mL) did not ablate hyper‐IL‐6‐induced *Sbno2* gene expression, indicating that the *Sbno2* gene is a primary response gene to the IL‐6/gp‐130 signaling pathway. Differences in Sbno2 (**B**) and Socs3 (C) mRNA levels between controls and hyper‐IL‐6 treated primary astrocytes during translational inhibition by CHX determined by two‐way ANOVA and Tukey's post test, *P < 0.05, **P < 0.01, ***P < 0.001. (*n* = 5–6). Superinduction of Sbno2 (B) and Socs3 (C) mRNA levels due to CHX addition were determined by 2‐way ANOVA and Bonferroni post‐tests, ^#^ *P* < 0.05, ^##^ *P* < 0.01, ^###^ *P* < 0.001 (*n* = 5–6).

In a time course, low levels of the SBNO2 protein isoforms were detected in untreated cells (0 h, Fig. 5B,C). The level of the ∼160 and ∼165 kDa SBNO2 proteins increased at 2 h, reached significance at 6 h (13‐fold), and remained elevated up to 24 h, when compared with untreated controls (Fig. 5B,C). Phospho‐STAT3 levels were very low in untreated (0 h) control astrocytes but increased and peaked (31‐fold) at 2 h after hyper‐IL‐6 (25 ng/mL) treatment and decreased thereafter (Fig. 5B,D). A significant increase in the ∼160 and ∼165 kDa SBNO2 proteins was also observed after treatment for 6 h with IL‐1β (5.9‐fold) and TNFα (6.4‐fold) (Fig. 5E,F). While no significant phosphorylation of ERK was observed in these cells (Fig. 5H), STAT3 phosphorylation was increased significantly by hyper‐IL‐6 (5.5‐fold, Fig. 5G) but not by IL‐1β or TNFα. Conversely, NFκB phosphorylation was increased significantly by IL‐1β (3.8‐fold) and TNFα (3.2‐fold) but not hyper‐IL‐6 (Fig. 6I) while the total STAT3, ERK, and NFκB levels did not change following treatment with any cytokine. In all, these findings illustrate that similar to the mRNA, the SBNO2 protein is increased significantly in astrocytes in response to hyper‐IL‐6, IL‐1β, and TNFα and in the case of IL‐1β and TNFα, can occur in the absence of STAT3 activation.

### *Sbno2* Is a Primary Response Gene to Hyper‐IL‐6 and Elevated *Sbno2* mRNA Levels Are Largely Dependent on Increased Transcriptional Activity

To investigate whether *Sbno2* gene expression is directly induced by the IL‐6/gp130 signaling pathways or indirectly as a secondary response to another protein that is induced by IL‐6/gp130, we treated astrocytes with hyper‐IL‐6 +/− the protein synthesis inhibitor cycloheximide (CHX, 10 μg/mL) (Fig. 6) for 2, 4, 6, and 8 h. At each treatment time, CHX significantly enhanced the effect of hyper‐IL‐6 upregulation of the Sbno2 (Fig. 6A,B) and Socs3 (Fig. 6A,C) mRNAs. While control groups did not differ significantly (Fig. 6A–C), the superinduction by CHX caused an increase of Sbno2 mRNA at 2 h (2.2‐fold) that peaked (8‐fold) at 6 h of treatment and remained elevated (4.9‐fold) up to 8 h (Fig. 6B, indicated by #). In contrast, Socs3 mRNA levels increased more rapidly and peaked (9‐fold) at 2 h with levels remaining elevated (8.6‐fold) up to 8 h compared with without CHX (Fig. 6C, indicated by #). In all, these results indicated that new protein synthesis was not required for hyper‐IL‐6‐stimulated *Sbno2* and *Socs3* gene expression.

**Figure 7:**
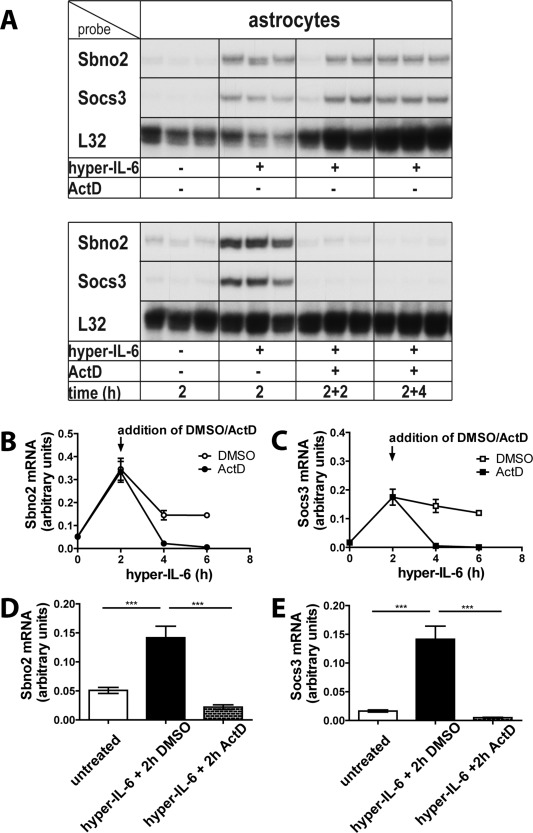
Sbno2 and Socs3 mRNAs are short‐lived and tightly regulated. Actinomycin D treatment (10 μg/mL) caused a depletion of hyper‐IL‐6‐ (25 ng/mL) induced Sbno2 and Socs3 mRNA levels within 2 h, indicating a rapid loss when new RNA synthesis is inhibited. **A**: A representative RPA is shown. At 2 h after inhibitor addition, Sbno2 (**B**) and Socs3 (**C**) mRNA levels decreased to control levels suggesting a high dependence on new RNA synthesis. **D**, **E**: Graphs show mean ± SEM of selected treatment groups from two individual RPAs with 2–3 samples per group (total *n* = 5–6). *P < 0.05, **P < 0.01, ***P < 0.001 compared by ANOVA and Tukey's post test.

To investigate the stability of the Sbno2 and Socs3 mRNAs during hyper‐IL‐6 signaling, we co‐treated cells with the inhibitor of transcription, actinomycin D (ActD). Primary astrocytes were treated with hyper‐IL‐6 and after a further 2 h, ActD or the vehicle DMSO alone was added (Fig. 7A–C). In astrocytes, 2 h after addition of ActD (i.e., 4 h total hyper‐IL‐6 treatment), Sbno2 mRNA (Fig. 7A,D) and Socs3 mRNA (Fig. 7A,E) levels were profoundly reduced to control levels. These findings suggest that the Sbno2 and Socs3 mRNAs are highly labile, rapidly being degraded in the absence of ongoing transcription.

### *Sbno2* mRNA Is Upregulated in the Murine Brain During Endotoxin‐Induced Systemic Inflammation and Is Localized to Astrocytes As Well As Other Cells

**Figure 8:**
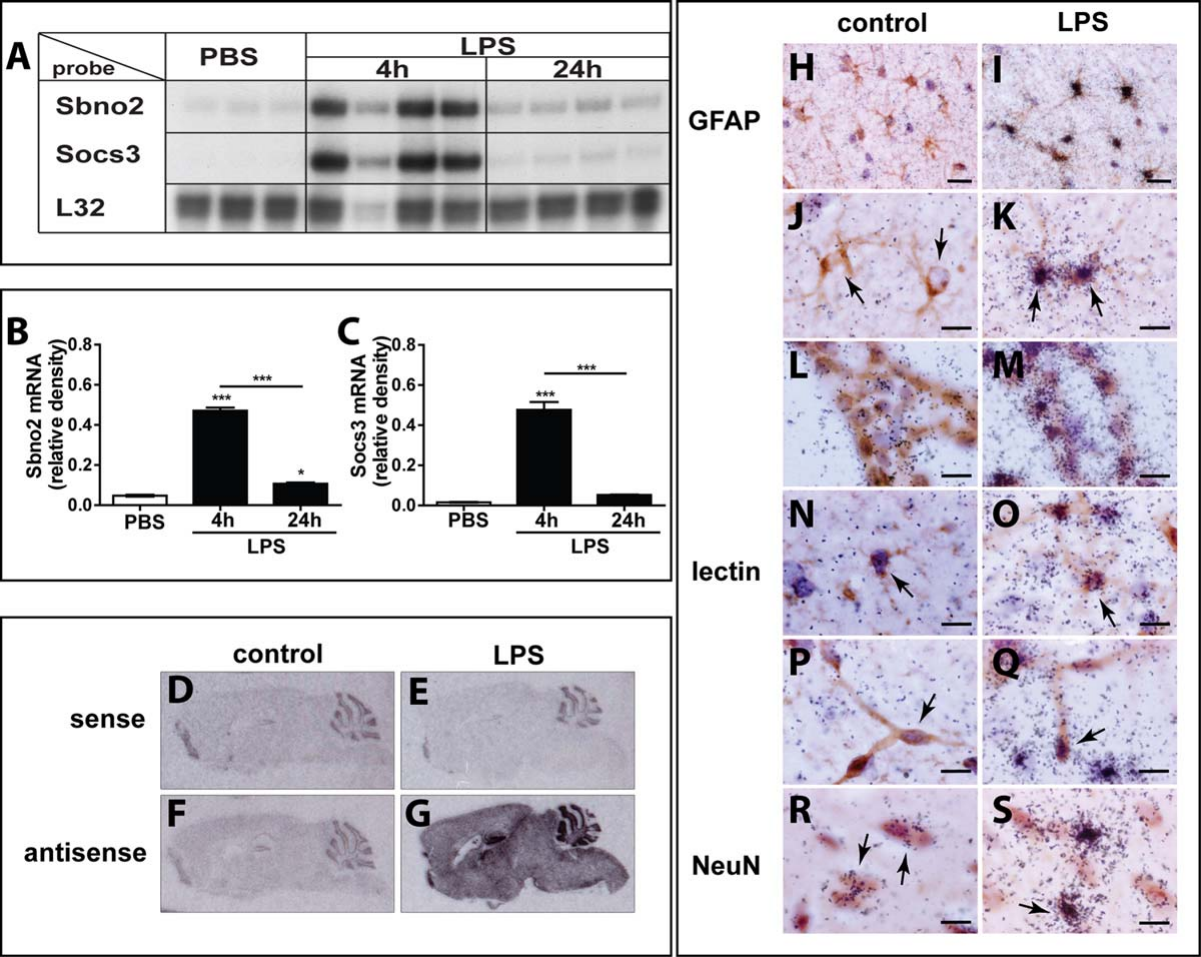
Sbno2 and Socs3 mRNAs are upregulated in the murine brain during endotoxin‐induced systemic inflammation and Sbno2 mRNA is located predominantly in astrocytes and the choroid plexus. **A**–**C**: Endotoxin‐induced inflammation caused a significant increase in Sbno2 (**B**) and Socs3 (C) mRNA in the murine brain at 4 h after the second LPS treatment. At 24 h, Sbno2 mRNA decreased but remained elevated compared with controls, while Socs3 mRNA returned to control levels. A: A representative RPA is shown. Graphs show mean ± SEM of treatment groups with 3–4 animals per group (total *n* = 3–4). *P < 0.05, **P < 0.01, ***P < 0.001 compared by ANOVA and Tukey's post test. **D**–**G**: ISH images of sense (D, **E**) and antisense (**F**, G) probe hybridization to tissue samples of control (D, F) and LPS‐treated (E, G) animals. ISH revealed a large increase in *Sbno2* mRNA throughout the brain during endotoxin‐induced inflammation (G) compared with controls (D–F). Tissue hybridized with sense probe showed non‐specific hybridization to the hippocampus, dentate gyrus, and cerebellar granule layer. No overt difference was seen in antisense control (F) compared with sense probe samples (D, E). **H, I**: Overview and (**J**–**S**) high magnification images of brain tissue sections from control (H, J, **L**, **N**, **P**, **R**) and LPS‐treated animals (I, **K**, **M**, **O**, **Q**, S). Astrocytes in controls only showed background hybridization (H), while after LPS treatment, almost all GFAP‐positive cells showed strong hybridization with the antisense probe (I). High magnification images (J–S) revealed strong hybridization of the Sbno2 antisense RNA probe to astrocytes (K, arrows) and cells of the choroid plexus (M) after LPS treatment. Some microglia (O, arrow), endothelial cells (Q, arrow), and neurons (S, arrow) showed an increase in Sbno2 mRNA hybridization after LPS treatment. Scale‐bar: 20 μm for H, I, 10 μm for J–S.

We next asked whether the expression of the *Sbno2* gene was regulated *in vivo* in a systemic endotoxemia model in which mice were given two staggered intraperitoneal injections of LPS (Ip et al., 2011; Stalder et al., 1997). In vehicle‐treated mice, there was low constitutive expression of the Sbno2 mRNA and even lower expression of the Socs3 mRNA (Fig. 8A–C). Following LPS injection, Sbno2 (9.9‐fold) and Socs3 (29‐fold) mRNA levels were increased markedly in the brain at 4 h after the second injection of LPS but decreased thereafter. Sbno2 mRNA was still significantly elevated in the brain at 24 h after the second LPS injection (2.3‐fold). These results showed that systemic LPS injection mediated significant stimulation of *Sbno2* and *Socs3* gene expression in the brain.

To investigate the source of the increased *Sbno2* gene expression in the murine brain following systemic LPS injection, we performed combined ISH‐IHC. Brain sections from control or LPS‐treated animals were hybridized with Sbno2 sense (Fig. 8D,E) or Sbno2 anti‐sense (Fig. 8F,G) probes. No appreciable difference was seen for hybridization signal between the Sbno2 anti‐sense control (Fig. 8F) and sense probes (Fig. 8D,E) in control brain sections. In brain sections from LPS‐treated mice (Fig. 8G), hybridization with Sbno2 anti‐sense probe revealed that Sbno2 mRNA was highly increased throughout the brain. Microscopic examination of LPS‐treated (Fig. 8I,K,M) mice compared with controls (Fig. 8H,J,L) revealed co‐localization of Sbno2 mRNA predominantly with GFAP‐stained astrocytes (Fig. 8I,K) and cells of the choroid plexus (Fig. 8M). At a lower magnification (Fig. 8I), nearly all astrocytes in brain sections from LPS‐treated mice were positive for Sbno2 mRNA which was further verified at higher magnification (Fig. 8K). In addition, selected tomato lectin‐stained microglia (Fig. 8O) and endothelial cells (Fig. 8Q) were also positive for Sbno2 mRNA. Finally, NeuN stained neurons showed co‐localization with low constitutive expression of Sbno2 mRNA in control tissue (Fig. 8R, arrows). After LPS administration, a small number of NeuN‐positive cells also were positive for Sbno2 cRNA hybridization (Fig. 8S, black arrow).Together, these findings showed that following systemic LPS challenge, *Sbno2* gene expression is markedly upregulated predominantly in astrocytes and the choroid plexus in the brain and to a lesser extent in some microglia, vascular endothelium, and neurons.

## Discussion

The pleiotropic cytokine IL‐6 and other members of the gp130 family are known inducers of astrogliosis (Campbell et al. 1993; Chiang et al. 1994; Damiani and O'Callaghan, 2007; Sriram et al., 2004). To better understand the impact of IL‐6 on astrocytes, DNA microarray analysis was performed on RNA from hyper‐IL‐6‐treated murine astrocytes and based on the resulting data (Frausto and Campbell, unpublished), the regulation of the *Sbno2* gene was further investigated here *in vitro* as well as *in vivo*. Our studies not only confirmed the initial microarray finding that hyper‐IL‐6 is a potent stimulus for *Sbno2* gene expression but showed that a number of other cytokines including those belonging to the IL‐6/gp130 family as well as IL‐1β and TNFα upregulate *Sbno2* gene expression. It had been reported previously that IL‐10 stimulates the expression of the *Sbno2* gene in BMDMs in a STAT3‐dependent manner (El Kasmi et al., 2007). Our results clearly showed that in astrocytes *Sbno2* gene expression is also upregulated by a number of different cytokines and suggest that, in addition to STAT3, other signaling factors such as NFκB are likely involved in the regulation of the *Sbno2* gene. Although it was originally suggested that Sbno2 played an important role in the IL‐10 AIR in BMDMs (El Kasmi et al., 2007), our findings here are consistent with the notion that Sbno2 has more wide‐ranging functions in the host response.

Similar to IL‐6, OSM, LIF, and IL‐11 are pleiotropic cytokines that exert pro‐ as well as anti‐inflammatory effects depending on the cellular microenvironment (Mathieu et al., 2012; Richards, 2013). In complex with gp130, the OSM and LIF receptors (OSMR, LIFR) are signaling receptors. However, this is not the case for the IL‐11 and IL‐6 receptors which rely entirely on gp130 for signal transduction. All the IL‐6/gp130 cytokines examined were able to induce Sbno2 mRNA levels in astrocytes with varying degrees of potency, but only OSM and hyper‐IL‐6, the strongest inducers of Sbno2 mRNA, also significantly induced Socs3 mRNA. When examining the dose‐response of astrocytes to hyper‐IL‐6, EC50 values for hyper‐IL‐6 induction differed considerably between the *Sbno2* and *Socs3* genes being much lower for *Sbno2* compared with *Socs3*. Therefore, while weaker stimuli might be potent enough to induce expression of the *Sbno2* gene, this may not achieve a threshold required to induce *Socs3* gene expression.

We showed here that upregulation of Sbno2 and Socs3 mRNA levels by IL‐1β and TNFα was associated with phosphorylation of NFκB p65 but not ERK1/2 or STAT3 which suggested an involvement of NFκB in regulation of both the Sbno2 and *Socs3* genes. However, the precise mechanism involved in the regulation of *Sbno2* gene expression by IL‐1β and TNFα remains to be determined.

Using a C‐terminal‐targeted anti‐SBNO2 antibody, we identified by Western blot both a constitutive and a cytokine‐induced isoform of the SBNO2 protein. The specificity of the primary antibody used in these studies was confirmed by incubation of the antibody with the immunizing blocking peptide which completely ablated reactivity with these proteins. Hutchins et al. (2012) showed previously that *Sbno2* belongs to a class of genes which undergo isoform switching. This is defined as an event where genes can switch to a new dominant transcription start site or a different splice isoform upon stimulation (Hutchins et al., 2012; Trapnell et al., 2010). A putative alternative promoter site controlling the transcription of the *Sbno2* gene short isoform contains a STAT3 binding site as demonstrated by CHIPseq analysis in macrophages (Hutchins et al., 2012). Assuming that the transcripts only differ in their N‐terminal end, as both can be detected by our C‐terminal‐targeted antibody, the calculated sizes from unmodified amino acid sequences are 141 kDa (NCBI Accession: XP_006513560.1) and 149 kDa (NCBI Accession: NP_906271.1), respectively. We detected a short, regulated double protein at ∼160–165 kDa, and a second, bigger constitutive protein at ∼175 kDa. The size difference between the two transcripts fits to the predicted size difference. Slightly higher apparent molecular weights can likely be caused by either splice differences or post‐translational modifications. Multiple species detected by immunoblot can be caused by one transcript but different modification states, e.g. differences in phosphorylation.

Comparing the two putative primary transcripts that encode the SBNO2 isoforms, there is no obvious loss of key functional domains between the long or the short forms of SBNO2. According to the alternative transcription start site, the short transcript has an alternative N‐terminal sequence that lacks 74 amino acids of the long transcript and differs by another 15 amino acids which does not affect the conserved helicase domains described previously in other Sno homologs (Simms and Baillie, 2010; Takano et al., 2010).

CHX treatment showed that induction of *Sbno2* and *Socs3* gene expression upon hyper‐IL‐6 treatment does not require any further protein synthesis. Both Sbno2 and Socs3 mRNA transcripts were superinduced upon CHX treatment with differences in their time courses. Addition of the transcriptional inhibitor actinomycin D to hyper‐IL‐6‐treated astrocytes revealed a rapid reduction in both Sbno2 and Socs3 mRNA transcripts. The short half‐life of these RNAs following blockade of transcriptional activity is consistent with there being a rapid degradation and tightly‐controlled regulation of these mRNAs. Furthermore, these data suggest that due to CHX translational inhibition, the synthesis of a protein or proteins that negatively regulate Sbno2 and Socs3 mRNAs may be prevented. One such inhibitor of the IL‐6/gp130 pathway is SOCS3 which inhibits gp130 signaling (Croker et al., 2008, 2003). While *Sbno2* and *Socs3* gene expression are similarly affected by the missing inhibitory effect due to the absence of SOCS3 protein, a higher increase of Socs3 mRNA was expected due to its higher inducibility with increasing doses (see dose‐response). Nevertheless, the very strong and rapid increase at 2 h, as well as the difference in kinetics of Sbno2 mRNA regulation compared with that for Socs3 mRNA following CHX treatment, suggest that additional mechanisms are likely involved regulating the stability of these mRNAs.

Our *in vivo* study clearly showed a significant increase in *Sbno2* gene expression in the murine brain and particularly in astrocytes following two staggered systemic LPS injections. This dual‐LPS injection model for endotoxemia has been shown previously to result in a pronounced increase in the expression of a number of pro‐inflammatory cytokine genes including IL‐1β, IL‐6, and TNFα (Ip et al., 2011; Stalder et al., 1997). Here, we have shown *in vitro* that all of these cytokines are able to induce *Sbno2* gene expression in astrocytes. While the precise factors involved in the upregulation of *Sbno2* gene expression in the LPS‐stimulated brain of mice remains to be determined, it is likely that IL‐1β, IL‐6, and TNFα acting alone or together contribute to this process.

More studies are needed to determine the precise function of SBNO2. However, in general, most genes with mRNAs with very short half‐lives were shown to encode for proteins with regulatory functions (Sharova et al., 2009). While the biological importance of SBNO2 remains unclear it was commented that complete knockout of the *Sbno2* gene is embryonic lethal (El Kasmi et al., 2007) suggesting a crucial role in mammalian development. The low, constitutive expression of one SBNO2 isoform in astrocytes is consistent with an involvement in homeostatic gene regulation. Upon stimulation with inflammatory factors and possible isoform switching, we have shown that a cytokine‐inducible form of SBNO2 is produced. All the cytokines that we have studied here have a positive effect on *Sbno2* gene regulation and were previously described to induce astrogliosis. Furthermore, sno homologs and SBNO2 in particular were previously shown to interact with transcription factors and play a role as transcriptional regulators (Maruyama et al., 2013; Tsuda et al., 2002). The presence of a nuclear localization signal and sequence similarities to DExD/H‐box proteins of the SWI/SNF ATPase family would suggest the possibility of an involvement of SBNO2 in chromatin remodeling processes. Consequently, SBNO2 could be involved in the regulation of transcriptional changes during induction and/or maintenance of an activated astroglial phenotype; however, this remains to be resolved.

In summary, our study has established for the first time that the upregulation of the *Sbno2* gene occurs in astrocytes as a direct response to a variety of different classes of cytokines. Moreover, we have shown that *Sbno2* gene expression is increased markedly in the brain in different cell‐types following LPS‐induced systemic endotoxemia, with astrocytes representing a major cellular source of *Sbno2* gene expression. These findings greatly extend our current understanding of the cellular distribution and regulatory control of the *Sbno2* gene in mammals and implicate SBNO2 as a key factor in the host response particularly in neuroinflammatory states.
